# Flunarizine suppresses endothelial Angiopoietin-2 in a calcium - dependent fashion in sepsis

**DOI:** 10.1038/srep44113

**Published:** 2017-03-09

**Authors:** Jennifer Retzlaff, Kristina Thamm, Chandra C. Ghosh, Wolfgang Ziegler, Hermann Haller, Samir M. Parikh, Sascha David

**Affiliations:** 1 Division of Nephrology and Hypertension, Hannover Medical School, Germany; 2 Beth Israel Deaconess Medical Center and Harvard Medical School, Center for Vascular Biology Research, Boston, MA, USA; 3 Department of Pediatric Kidney, Liver and Metabolic Diseases, Hannover Medical School, Germany.

## Abstract

Sepsis is a life-threatening organ dysfunction caused by a dysregulated host response to an infection leading to systemic inflammation and endothelial barrier breakdown. The vascular-destabilizing factor Angiopoietin-2 (Angpt-2) has been implicated in these processes in humans. Here we screened in an unbiased approach FDA-approved compounds with respect to Angpt-2 suppression in endothelial cells (ECs) *in vitro*. We identified *Flunarizine* – a well-known anti-migraine calcium channel (CC) blocker – being able to diminish intracellular Angpt-2 protein in a time- and dose-dependent fashion thereby indirectly reducing the released protein. Moreover, Flunarizine protected ECs from TNFα-induced increase in Angpt-2 transcription and vascular barrier breakdown. Mechanistically, we could exclude canonical Tie2 signalling being responsible but found that three structurally distinct T-type - but not L-type - CC blockers can suppress Angpt-2. Most importantly, experimental increase in intracellular calcium abolished Flunarizine’s effect. Flunarizine was also able to block the injurious increase of Angpt-2 in murine endotoxemia *in vivo*. This resulted in reduced pulmonary adhesion molecule expression (intercellular adhesion molecule-1) and tissue infiltration of inflammatory cells (Gr-1). Our finding could have therapeutic implications as side effects of Flunarizine are low and specific sepsis therapeutics that target the dysregulated host response are highly desirable.

Sepsis is a severe but under-recognized disease affecting millions of people every year worldwide. Its definition has very recently been updated for the third time as a life-threatening organ dysfunction caused by a dysregulated host response to infection^1^,^2^. In fact, its incidence appears to be rising and the mortality ranges between 38 and 59%^3^. The lack of specific therapies is not for want of effort, but probably also a result of focusing almost exclusively on elucidating highly complex humoral and immune pathways incited by bacteria and their products. Monitoring this complex and redundant system is hard to achieve. From a therapeutic standpoint, what may save one patient may harm another.

While the immune system is undoubtedly important in the pathogenesis, somewhat less attention has been given to the microvasculature in sepsis where the endothelium presents a pro-coagulant and pro-adhesive surface, fails to produce its usual profile of vasoconstrictive and vasodilatory compounds, and suffers a loss of normal barrier function^4^. Of these changes, increased vascular permeability may be particularly important because it gives rise to hypovolemia, contributes to hemo-concentration, stasis of blood flow, and shock^5^. Interestingly, the new sepsis definition moves away from the central role of inflammation towards organ dysfunction and highlights the critical importance of the overwhelming host response. the systemic endothelial dysfunction in sepsis is a fundamental part of this dysregulated host response.

The Angiopoietin (Angpt)/Tie2 ligand receptor system controls baseline endothelial function with respect to inflammation, survival and vascular barrier function via continuous Angpt-1 driven Tie2 phosphorylation^6^. In sepsis, the endothelium releases high levels of pre-stored Angpt-2 - the natural antagonist of the Tie2 receptor – thereby inducing inflammation and vascular barrier breakdown actively contributing to multiple organ dysfunction and death^7^,^8^. Consistently, the circulating Angpt-1/Angpt-2 homeostasis in septic patients is severely shifted in favour for Angpt-2^9^. Therapeutic modulation of this ligand-receptor system could represent a promising innovative strategy against the capillary leakage syndrome of highly septic individuals^10^. In classical proof of principle studies, we and others have used genetic Angpt-2 depletion or siRNA treatment as well as a synthetic Tie2 agonistic peptide to successfully demonstrate the therapeutic potential of this concept^8^,^11^,^12^,^13^,^14^.

To overcome the immense difficulties in drug development and the consecutive translational gap from bench to bedside, we screened FDA-approved small molecules in an unbiased library approach for their ability to reduce Angpt-2 in endothelial cells *in vitro*. We identified a long-known anti-migraine calcium channel (CC) blocker termed Flunarizine and analysed its molecular mechanism of action *in vitro* and translated these findings in a murine *in vivo* scenario. In summary, we found evidence that Flunarizine might lower Angpt-2 in a calcium-dependent fashion *in vitro* and *in vivo*.

## Results

### FDA-library screening identifies ‘Flunarizine’ as a potent inhibitor of Angpt-2 production

**Figure 1:**
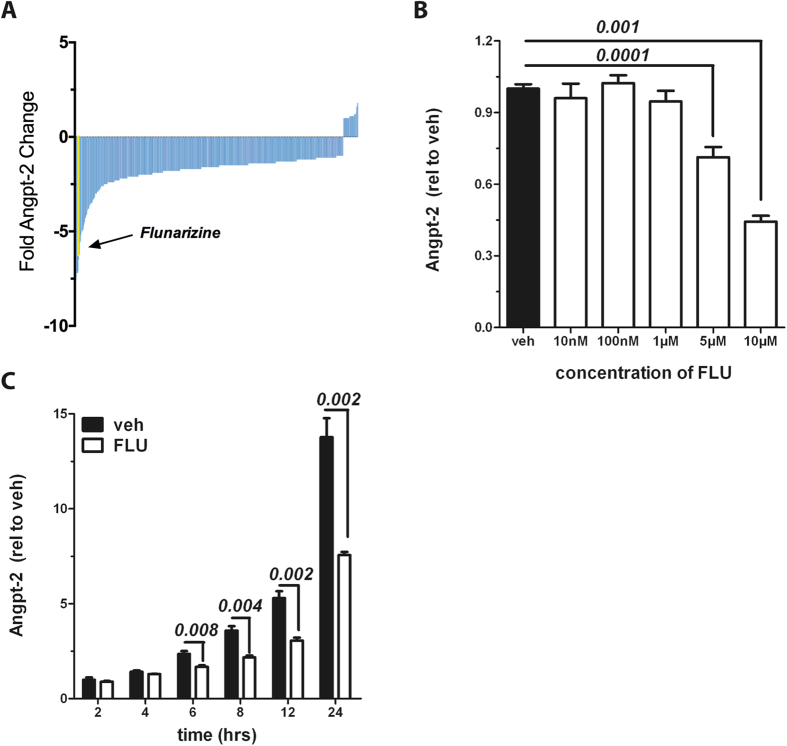
Flunarizine reduces baseline Angiopoietin-2 (Angpt-2) *in vitro.* (**A**) Human umbilical vein endothelial cells (HUVECs) grown in a 96-well format were treated with a Food and Drug Administration (FDA)-drug library for 24 hrs, and Angpt-2 protein in the supernatant was measured by enzyme-linked immunosorbent assay (ELISA). Results were analyzed as the fold-change relative to the median value and ordered from strongest inhibitors (left, blue bars) to strongest inducers (right, blue bars). The cutoff was set to tenfold reduction. (**B**) HUVECs were treated with different concentrations of Flunarizine for 24 hrs and Angpt-2 in the supernatant was quantified by ELISA (n = 6–12). (**C**) 10 μM Flunarizine (FLU) or vehicle was applied for indicated time points. Angpt-2 in the supernatant was measured by ELISA (n = 5–6). Columns are presented as mean ± SEM.

Potentially injurious Angpt-2 is stored and secreted by endothelial cells (ECs) in the septic human organism thereby contributing to disease morbidity and mortality^8^. To test various pharmaceuticals regarding their potency to lower Angpt-2, we applied a library of FDA-approved small molecules^15^ at a standardized dose for 24 hrs to HUVECs and measured Angpt-2 in the supernatant (Fig. 1A). Most drugs had no significant effect, but the anti-migraine drug Flunarizine profoundly decreased Angpt-2. Detailed *in vitro* studies with Flunarizine confirmed a potent dose-/ (Fig. 1B) and time-dependent (Fig. 1C) effect on Angpt-2 in the media of ECs. The most prominent effect was seen after 24 hrs with a 10 μM dose.

**Figure 2:**
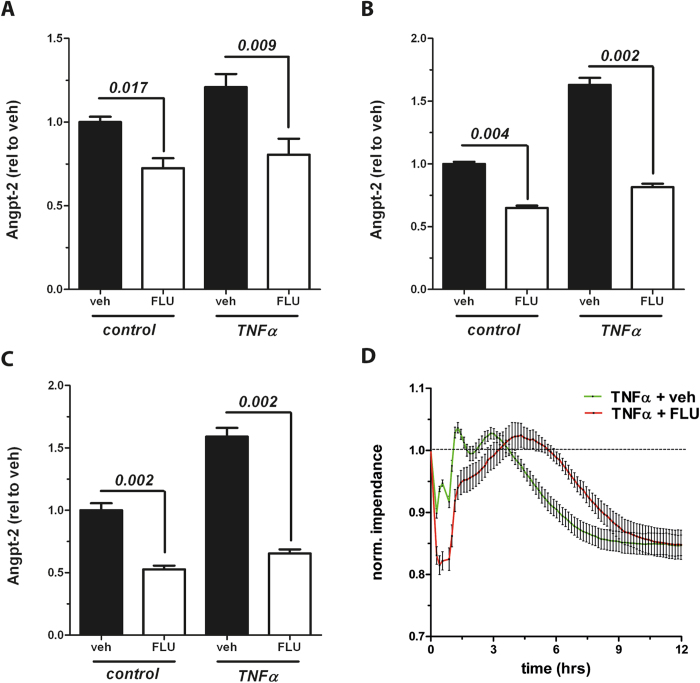
Flunarizine reduces Angiopoietin-2 (Angpt-2) after stimulation *in vitro*. Human umbilical vein endothelial cells (HUVECs) were stimulated with 10 ng/mL tumor necrosis factor α (TNFα) or control after 1 h pretreatment with either 10 μM Flunarizine (FLU) or vehicle and the concentration of Angpt-2 in the supernatant was determined by enzyme-linked immunosorbent assay (ELISA) (**A**) after six hrs of stimulation (n = 6) (**B**) after 12 hrs of stimulation (n = 6) and (**C**) after 24 hrs of stimulation (n = 6) (**D**) Real-time transendothelial electrical resistance (TER) from HUVECs, who were pretreated for 1 h with 10 μM Flunarizine (FLU) or vehicle and stimulated with 10 ng/mL TNFα, was recorded with an electric cell-substrate impedance sensing (ECIS) device (ibidi). Columns are presented as mean ± SEM.

In order to study the underlying mechanisms, we then tested the excessive release of Angpt-2 from ECs upon stimulation with various sepsis mediators (i.e. IL-1ß, TNFα, LPS, IFNγ, Supplemental Fig. 1). Of those, TNFα showed a strong and reproducible Angpt-2 induction. Therefore, we tested the effect of Flunarizine on Angpt-2 in TNFα stimulated ECs and found that Flunarizine completely blocked the accumulation of Angpt-2 in the media of challenged ECs indicating an even higher potential if ECs are stressed compared to baseline quiescence. This result was detectable as early as 6 hrs (Fig. 2A) and in the following time period after 12 and 24 hrs (Fig. 2B,C). We then evaluated the functional relevance of these findings with respect to endothelial permeability. To do this, we recorded real-time transendothelial electrical resistance (TER) with an electric cell-substrate impedance sensing (ECIS) device after stimulation with TNFα and found a delay in endothelial barrier breakdown in Flunarizine treated cells for 2.0 ± 0.6 hrs (Fig. 2D).

### Flunarizine influences endothelial Angpt-2 protein biosynthesis rather than exocytosis

**Figure 3:**
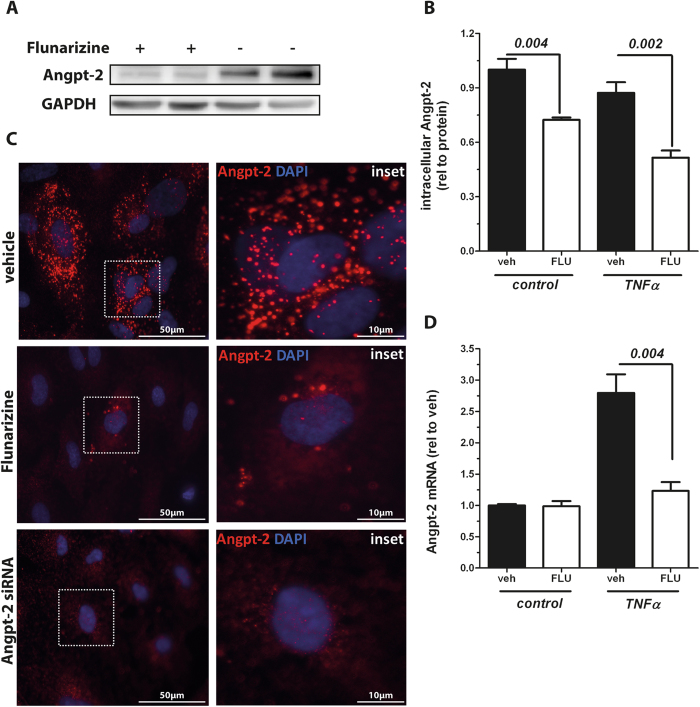
Flunarizine reduces Angiopoietin-2 (Angpt-2) synthesis *in vitro*. (**A**) Cropped Angpt-2 immunoblot from human umbilical vein endothelial cell (HUVEC) lysates 15 hrs after 10 μM Flunarizine (FLU) (+) or vehicle (−) treatment. (n = 4) (**B**) After stimulation with 10 μM FLU or vehicle for 1 h, 10 ng/mL Tumor necrosis factor α (TNFα) or control was applied to HUVECs for 24 hrs. Angpt-2 concentration in cell lysates was measured by enzyme-linked immunosorbent assay (ELISA) and is shown relative to whole protein (n = 6). (**C**) Fluorescent immunocytochemistry for Angpt-2 (red) and nuclear staining (4′,6-diamidino-2-phenylindole, blue) in HUVECs after treatment with 10 μM Flunarizine, vehicle or Angpt-2 siRNA as a negative control for 24 hrs (n = 4) **(D**) Real-time polymerase chain reaction (RT-qPCR) for Angpt-2 in HUVECs stimulated with 10 ng/mL TNFα or control for 15 hrs after pretreatment with either FLU or vehicle (n = 6) for 1 h. Columns are presented as mean ± SEM.

Given that the effect of Angpt-2 suppression was most prominent at late time points (Fig. 1C) we analysed if biosynthesis of Angpt-2 might be the primary target of Flunarizine’s action rather than solely the exocytosis of pre-stored protein. It is important to know that *in vitro* ECs continuously release Angpt-2 into the media (Fig. 1C, black bars). Consequently, it was unclear if Flunarizin affects intracellular protein and thereby indirectly the released one or if it mechanistically interacts with the process of exocytosis. We immunoblotted EC lysates for Angpt-2 to quantify the intracellular storage. Indeed, ECs that have been treated with Flunarizine showed less Angpt-2 within the cell lysates (Fig. 3A,B). Fluorescent immunocytochemistry for Angpt-2 confirmed our results as HUVECs that were treated with Flunarizine did clearly contain less Angpt-2 compared to control treated cells. Vehicle treated cells had their Weibel-palade bodies (specialized granules that store Angpt-2, vWF and various cytokines) impressively filled with pre-stored Angpt-2 ready for release upon endothelial activation (Fig. 3C). Again, if Flunarizine would inhibit Angpt-2 exocytosis one would expect analogously (over-)filled granules. To test if transcription might be involved we then performed quantitative Angpt-2 RT-PCRs at baseline and after TNFα stimulation. Indeed, Flunarizine significantly reduced the otherwise elevated Angpt-2 transcription upon stimulation (Fig. 3D and Supplemental Fig. 2A). However, baseline Angpt-2 mRNA was not affected by Flunarizine, potentially indicating another posttranscriptional mechanism of action. Having used TNFα as a stimulator we tested Flunarizine’s effect on TNFα signalling and found reduced canonical downstream activation (i.e. phosphorylation of JNK, Supplemental Fig. 2B). On the other hand, Flunarizine was still effective in Angpt-2 lowering if ECs where challenged with other mediators than TNFα (e.g. Interleukin(IL)-1β, Supplemental Fig. 2C) suggesting that TNFα signaling cannot entirely explain this observation. Together these data indicate that Flunarizine’s baseline effect on Angpt-2 rather depends on posttranscriptional events than on influencing exocytosis. To the contrary, upon stimulation (i.e. TNFα) Flunarizine prevents increase of Angpt-2 upstream of transcription.

### Flunarizine does not require Tie2 or downstream signalling to reduce Angpt-2

Angpt-1 ligation to Tie2 leads to its phosphorylation (i.e. activation) and negatively affects Angpt-2 transcription in the sense of an inhibitory feedback loop^16^. We therefore tested if Flunarizine’s negative effect on Angpt-2 is driven by an up-stream regulation of the Tie2 receptor.

**Figure 4:**
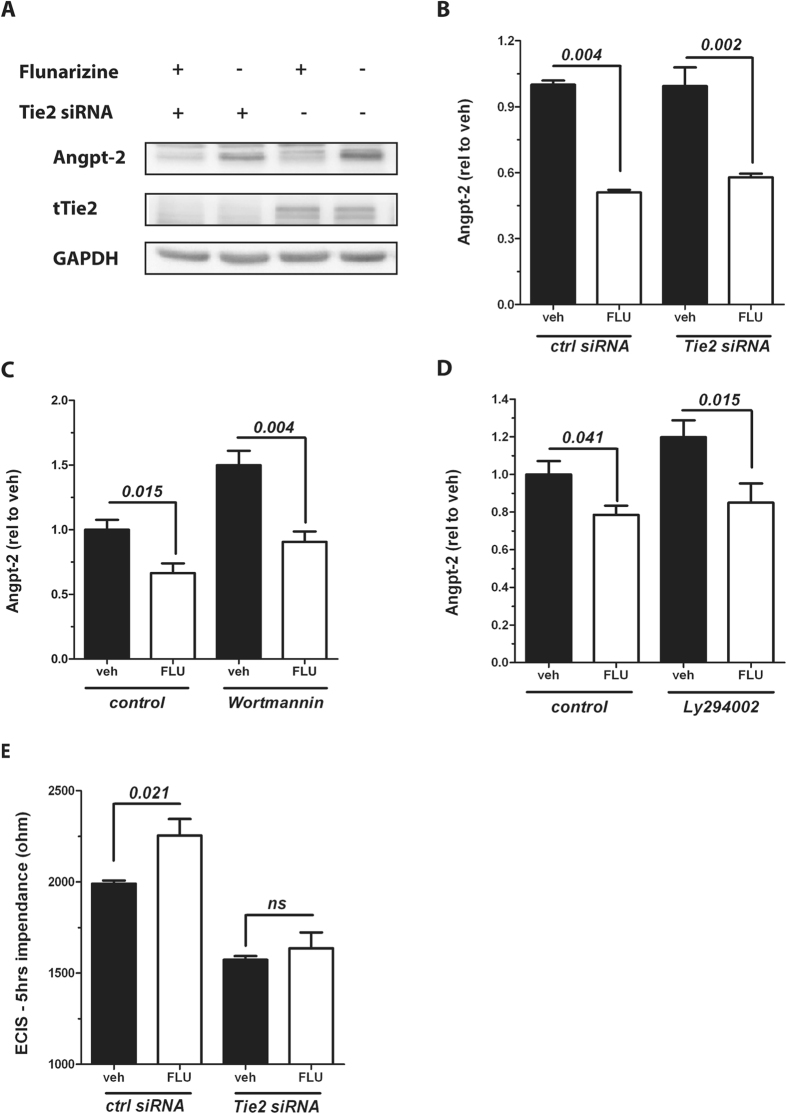
Flunarizine does not require Tie2 or downstream signalling to reduce Angpt-2. (**A**) Cropped immunoblot for Angiopoietin-2 (Angpt-2), tTie2 and GAPDH as a loading control from HUVECs transfected with control siRNA or Tie2 siRNA and stimulated with 10 μM Flunarizine (FLU) or vehicle for 24 hrs (n = 4). (**B**) HUVECs were treated with 10 μM FLU (+) or vehicle (−) for 24 hrs after transfection with Tie2 siRNA (+) or control siRNA (−) and the concentration of Angpt-2 in the supernatant was determined by ELISA (n = 5–6). Angpt-2 concentration in the supernatant of cells pretreated with (**C**) 1 μM Wortmannin or control for 1 h or with (**D**) 50 μM LY294002 for 1 h and stimulated with 10 μM FLU or vehicle for 12 hrs was measured by enzyme-linked immunosorbent assay (ELISA) (n = 6). (**E**) Real-time transendothelial electrical impedance from HUVECs 24 hrs after transfection with Tie2 siRNA or control siRNA, who were pretreated with 10 μM Flunarizine (FLU) or vehicle for 1 h and stimulated with 10 ng/mL TNFα, was recorded with an electric cell-substrate impedance sensing (ECIS) device (ibidi). Bar graphs represent n = 8 measurements per conditions after 5 hrs of TNF challenge. Columns are presented as mean ± SEM.

We performed RNA interference (RNAi) experiments targeting Tie2. First of all, knockdown of Tie2 did not change intracellular Angpt-2 expression per se (Fig. 4A, lane 2 and 4) and had also no influence on Flunarizine-driven Angpt-2 suppression (lane 1 and 2). This finding was confirmed by ELISA from media from Tie2 silenced ECs+/− Flunarizine for 6 and 24 hrs (Fig. 4B and Supplemental Fig. 3A). To further elucidate if canonical Tie2 signalling is required for Flunarizine’s effect on Angpt-2 we pharmacologically blocked downstream AKT with *Wortmannin* and with *LY294002*. Consistent with the Tie2 siRNA experiment, downstream PI3K/AKT blockade did not abolish Flunarizine’s Angpt-2 lowering effect (Fig. 4C,D and Supplemental Fig. 3B,C). Moreover, Flunarizine did neither phosphorylate Tie2 (Y1100/1102) nor downstream AKT (Supplemental Fig. 3D). Next, we analysed if Tie2 – as the classical Angpt-2 receptor – is required for Flunarizine’s vascular protective properties. Using the ECIS device in Tie2 and control siRNA treated cells we found that Flunarizine lost his barrier protective properties if Tie2 was silenced (Fig. 4E). In conclusion, we could not demonstrate that Flunarizine’s Angpt-2 lowering effect is dependent on Tie2 but that the presence of Tie2 is required for functional improvement as a consequence of reduced Angpt-2.

### Flunarizine might suppress Angpt-2 in a calcium-dependent fashion

**Figure 5:**
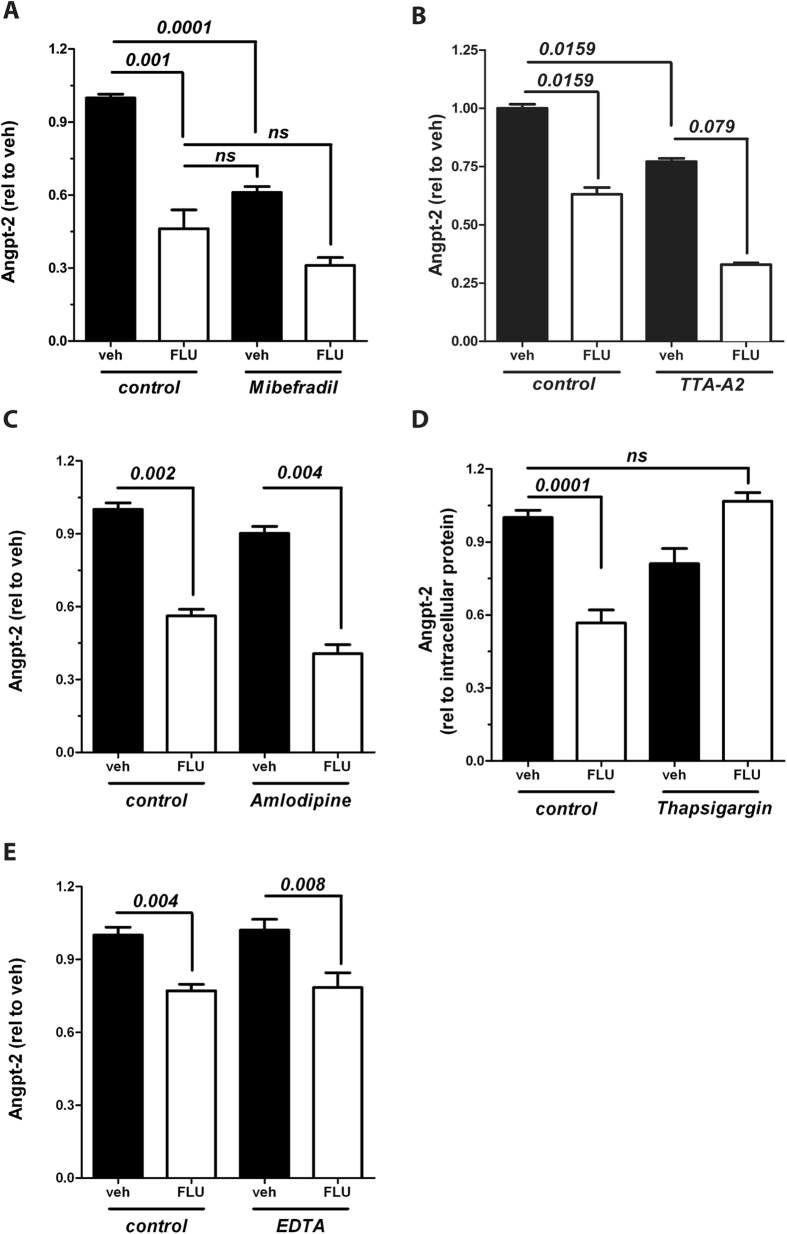
Multiple structurally distinct putative T-type (but not L-type) calcium channel blockers suppress Angiopoietin-2 (Angpt-2). 10 μM Flunarizine (FLU) or vehicle was applied for 24 hrs after pretreatment with (**A**) 10 μM Mibefradil or control for 1 h, (**B**) 50 μM TTA-A2 (a specific pharmacological inhibitor of t-type CCs) or control for 1 h, **(C)** 10 μM Amlodipine or control for 1 h. Angpt-2 concentration in the supernatant of human umbilical vein endothelial cells (HUVECs) was measured by enzyme-linked immunosorbent assay (ELISA) (n = 6–10). **(D)** Before application of 10 μM FLU or vehicle for 24 hrs HUVECs were pretreated with 1 μM Thapsigargin or control for 0.5 hrs. Angpt-2 concentration in the supernatant was measured by ELISA and is shown relative to whole intracellular protein (n = 6–10). (**E**) 10 μM FLU or control was applied for 8 hrs after pretreatment with 10 μM EDTA for 1 h and the concentration of Angpt-2 in the supernatant was determined by ELISA (n = 6). Columns are presented as mean ± SEM.

Given that Flunarizine’s effect as an anti-migraine drug is attributed to its properties as a CC blocker^17^ we hypothesized that cytosolic calcium concentration ([Ca^2+^]) might play a critical role in Angpt-2 synthesis and the interplay with Flunarizine. To test this, we analysed the Angpt-2 lowering capacity of other CC blockers. Flunarizine is considered a T-type CC blocker (although not absolutely specific). Interestingly, 2 other structurally distinct T-type CC blockers with different chemical and pharmacological properties^18^ (i.e. Mibefradil, Fig. 5A and TTA-A2^19^, Fig. 5B) nicely replicated the Angpt-2 suppression that we detected with Flunarizine. When we tested another CC blocker type (i.e. L-type) using Amlodipine, we could hardly detect any effect on Angpt-2 (Fig. 5C, lane 1 and 3). However, Flunarizine could still lower Angpt-2 in the presence of L-type CC blockade (lane 3 and 4). Speculating that calcium might be involved, we experimentally increased the cytosolic [Ca^2+^] by pharmacologically blocking the SERCA, a Ca^2+^ ATPase of the sarcoplasmatic reticulum (SR) that usually lowers cytosolic [Ca^2+^] by transporting it from the cytoplasm into the SR. To do so we used a drug called *Thapsigargin*. Indeed, in the presence of Thapsigargin, Flunarizine’s Angpt-2 lowering effect disappeared (Fig. 5D), indicating that intracellular calcium homeostasis might be important for Angpt-2 biology. Of note, when extracellular calcium was chelated using EDTA no such effect was recorded (Fig. 5E).

To our knowledge, a calcium-dependent mechanism of Angpt-2 regulation has not been reported yet. A canonical inductor of intracellular calcium signalling, i.e. PMA, induced a threefold increase in Angpt-2 (Supplemental Fig. 4A). Using life cell imaging and ECs transfected with a cytosolic calcium tracker, we found a weaker calcium influx upon TNFα stimulation when cells were pre-treated with Flunarizine (Supplemental Fig. 4B).

Although not proven, these findings indicate that Angpt-2 synthesis (and beneficial Flunarizine effects) could be modulated in a calcium-dependent fashion.

### Flunarizine lowers Angpt-2 transcription and circulating levels in murine endotoxemia and improves survival

**Figure 6:**
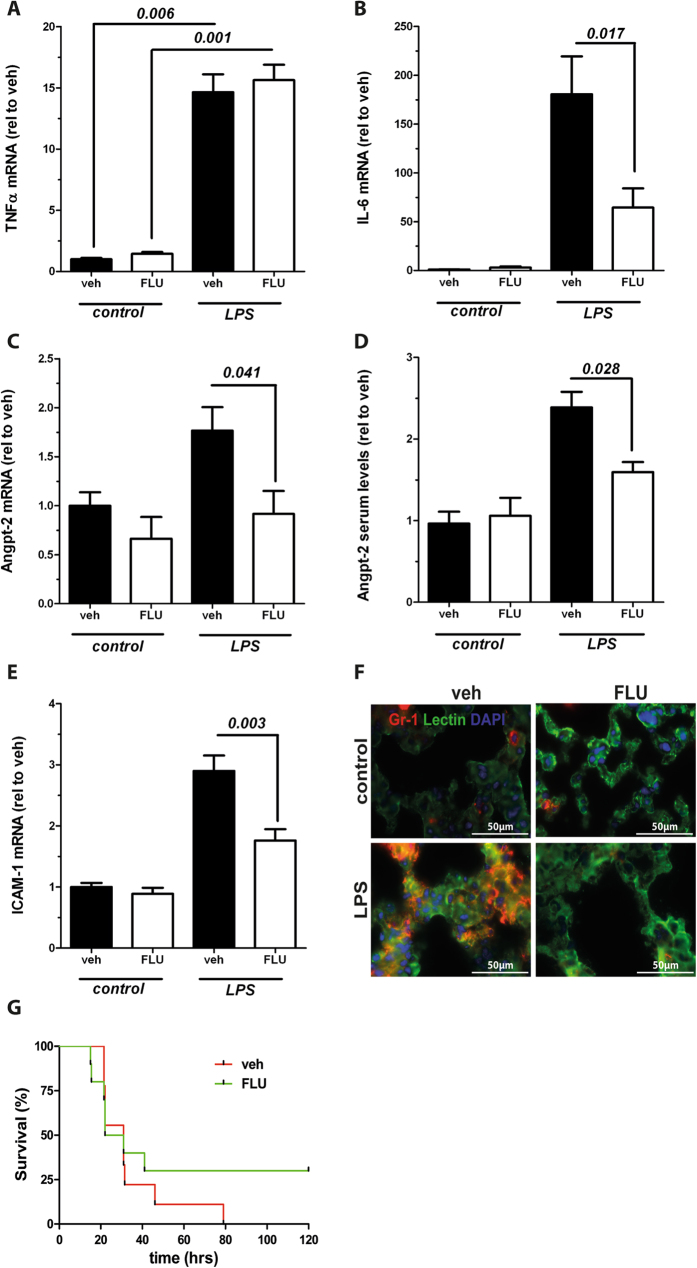
Flunarizine lowers Angiopoietin-2 (Angpt-2) and vascular inflammation *in vivo.* Adult male C57BL/6 J mice were pretreated daily with Flunarizine (25 mg/kg body weight (bw) p.o.) or vehicle for three days before the injection with 17.5 mg/kg bw gram-negative endotoxin (lipopolysaccharides [LPS] from Escherichia coli i.p.) or control. Expression of pulmonary mRNA and Angpt-2 serum levels were measured 12 h after LPS (n = 3–9). (**A**) Real-time polymerase chain reaction (RT-PCR) from lung homogenates for tumor necrosis factor α (TNFα). (**B**) RT-PCR from lung homogenates for Interleukin-6 (IL-6). **(C)** RT-PCR from lung homogenates for Angpt-2. (**D**) Angpt-2 serum levels were quantified by enzyme-linked immunosorbent assay (ELISA). (**E**) RT-PCR from lung homogenates for intercellular adhesion molecule-1 (ICAM-1). (**F**) Immunohistochemistry from murine lungs for Gr-1 (red), endothelial-specific lectin (green) and nuclear staining (4′,6-diamidino-2-phenylindole, blue) (**G**) Kaplan Meier Survival after LPS administration (20 mg/kg bw) in mice that were pretreated with 25 mg/kg bw FLU or vehicle for three days once a day (n = 8–10, p = 0.408). Columns are presented as mean ± SEM.

To analyse a putative translational relevance of our *in vitro* findings we next investigated the effect of Flunarizine on Angpt-2 in a murine endotoxemia model *in vivo*. Male C57Bl/6 J mice were challenged with LPS from *E. coli* (17.5 mg/kg BW i.p.) and organs were harvested after 12 hrs. A sepsis-like inflammatory response to LPS was confirmed by the expression of classical cytokines such as TNFα and IL-6 (Fig. 6A,B). Of those cytokines, IL-6 but not TNFα significantly reduced Flunarizine. Analogously to our *in vitro* findings, Flunarizine was able to significantly reduce Angpt-2 expression in murine lungs (Fig. 6C) but not in other organs with a lower amount of capillaries per tissue (e.g. kidneys, Supplemental Fig. 5). As shown before, circulating levels of Angpt-2 measured by ELISA in the murine serum were increased in endotoxemia. However, mice that were pre-treated with Flunarizine were protected from this injurious Angpt-2 increase (Fig. 6D).

Consistently, this reduction of Angpt-2 *in vivo* also reduced the expression of ICAM-1, an adhesion molecule well known to be regulated by Tie2 (Fig. 6E). Most likely as a direct consequence of reduced ICAM-1, we also observed less pulmonary infiltration of inflammatory cells, shown by fluorescent immunohistochemistry for Gr-1 and Lectin (Fig. 6F). To put these data in a clinically meaningful context we also performed a pilot survival study. Consistent with our hypothesis, we found that Flunarizine showed a trend towards improved survival by 30% (Fig. 6G, Kaplan Meier survival, p = 0.408).

## Discussion

Given the outrageous costs and the time consuming process of drug development in any medical field, novel unbiased approaches to re-assess “off-target” effects of already approved compounds are highly attractive. To our knowledge this is the first report demonstrating that the CC blocker Flunarizine is able to lower the vascular-destabilizing factor Angpt-2 both *in vitro* and *in vivo*. Given that excess Angpt-2 contributes to vascular leakage and multiple organ dysfunction (MOD) in mice and is associated with MOD and mortality in men^8^ this finding might have therapeutic implications for the future treatment of septic shock patients^10^,^20^. In general, causal treatment strategies that target the dysregulated host response side by side with the antimicrobial infection control are urgently required. Flunarizine might yield potential in regulating the overwhelming host response in particular with regard to vascular barrier breakdown by reducing injurious Angpt-2. From a mechanistic point of view this work suggests a potential calcium-dependent regulation of endothelial Angpt-2.

Flunarizine is a CC blocker with a high affinity (but not 100% specificity) to so-called T-type channels^21^ that is clinically used for prophylaxis of migraine^22^ and vertigo^23^ in contrast to the L-type blocking dihydropyridines (e.g. Amlodipine, among others). Those L-type blockers are highly effective drugs against arterial hypertension that are used on a daily routine basis in millions of people around the globe^24^. Flunarizine’s mode of action in migraine is still discussed. Ye *et al*. recently suggested its sodium and calcium channel blocking activity in cortical neurons as one possible explanation^25^. Furthermore, Flunarizine can inhibit norepinephrine-induced vasoconstriction in mesenteric arteries, also via a calcium-dependent mechanism^26^. Not only vascular tone is affected by Flunarizine, but there are also several endothelium-protective properties described; Flunarizine e.g. leads to less venostatic thrombosis, less endothelial damage induced by citrate-injection^27^, as well as less permeability^28^,^29^. Our findings contribute to the knowledge about the variety of Flunarizine’s beneficial pleiotropic effects on the endothelium and expand its importance by showing that Flunarizine-mediated suppression of Angpt-2 might be useful for the treatment of septic vascular barrier breakdown.

In this study, Flunarizine’s *in vitro* effects on Angpt-2 were highly reproducible in various inflammatory situations whereas the *in vivo* scenario was undoubtedly more complicated. In general, the solubility of Flunarizine in normal saline and DMSO is low. Therefore intravenous application is tedious and did not lower the Angpt-2 level neither in blood nor on the tissue level (data not shown). However, when given orally at a relatively high dose of 25 mg/kg BW we could nicely replicate the robust *in vitro* findings. With respect to the doses used in this study we have roughly calculated cross-species equivalent doses to demonstrate that the *in vitro* doses are comparable to the approved human anti-migraine dose.

Based on a well-known negative feedback loop on Angpt-2 via Tie2^16^ we have speculated that Flunarizine might require Tie2 signalling to suppress Angpt-2. The key role of Tie2 has recently been demonstrated by pharmaceutical approaches to modulate the activity of Tie2 in sepsis^30^ and acute kidney injury (AKI)^31^. However, the observation that both blocking canonical Tie2 signalling (i.e. PI3K/AKT) and genetically lowering Tie2 expression did not affect Flunarizine’s Angpt-2 lowering potential suggests a Tie2 independent effect.

We found that calcium might play a role in Flunarizine’s effect on Angpt-2 although we could not definitely demonstrate the responsible channels. Most importantly, the existence of T-type CCs in the endothelium has not been described. However, several leads point towards a calcium-dependent mechanism: (1) Canonical induction of intracellular calcium signalling, i.e. PMA, was sufficient to induce a threefold increase in Angpt-2. (2) Other structurally distinct T-type CC blocker (i.e. Mibefradil - admittedly also not 100% specific, but also another highly T-type selective CC blocker, termed TTA-A2) replicate Flunarizine’s effects on Angpt-2. Of note, TTA-A2 inhibits all three subtypes of low-voltage-gated T-type channels (Ca_v_3.1, Ca_v_3.2, and Ca_v_3.3) with comparable potencies^19^. (3) L-type CC blocker (i.e. Amlodipine) have no effect on Angpt-2 but do not affect Flunarizine’s additive effect. (4) Increased cytosolic calcium concentration by blocking SERCA - a pump that physiologically lowers cytosolic Ca^2+^ concentration by transporting it into the sarcoplasmic reticulum –abolished Flunarizine’s effect on Angpt-2. (5) RT-PCR screening at least suggests the endothelial expression of Ca_v_3.2 and Ca_v_3.3 (data not shown). (6) Qualitative data from life cell imaging experiments show a delayed calcium influx in ECs after Flunarizine stimulation. Together our data suggest a relevant role of calcium in endothelial Angpt-2 biology. Of course, we cannot exclude unknown off-target effects such as blockade of other CCs or even targets completely unrelated to calcium. However, a literature research did not reveal any common off-target effect of the three tested T-type CC blockers (Flunarizine, Mibefradil, TTA-A2). The observation that the Angpt-2 transcript was only reduced after TNFα stimulation (but not at baseline, Fig. 3D) together with reduced canonical TNFα downstream signalling (i.e. pJNK, Suppl. Fig. 2B) upon Flunarizin treatment might suggest an interaction with the TNFα receptor.

Our study has limitations. As mentioned above we can only speculate on the existence of an endothelial T-type CC. This study was not designed to prove CC expression in the endothelium but to detect FDA-approved drugs with Angpt-2 lowering potential. If Flunarizine’s effect is indeed mediated through calcium is still not clear and future studies are highly desirable to better understand the role of calcium in the regulation of Angpt-2. Second, the *in vivo* data are promising as a proof of principle but are based on an early pre-treatment strategy that is – at least with respect to sepsis – not transferable to human disease where patients present in the hospital with manifest disease symptoms and doctors can only administer drugs after the onset of disease.

Although basic research has to provide more evidence for the existence of vascular endothelial T-type CCs, various CC blockers are FDA-approved and have a well-known side effect profile. Therefore future studies that investigate their role in the treatment of Angpt-2 driven multiple organ dysfunctions in sepsis are very exciting and might be generally feasible.

## Methods

### Antibodies and Reagents

All chemicals and reagents were purchased from Sigma-Aldrich (St. Louis, MO) unless otherwise specified. Antibodies against Angpt-2 (AF623) (R&D Systems, Minneapolis, MN), mAngpt-2 (Clone #748246) (R&D Systems, Minneapolis, MN), Tie2 (C-20) (sc-324, Santa Cruz Biotechnology, CA), pTie2 (Y1102/Y1100) (R&D systems, Minneapolis, MN), Gr-1 (AbD Serotec), Lcyopersicon esculentum agglutinin (LEA, tomato lectin) (Vector Laboratories, Burlingame, CA), Akt (11E7) (Cell Signaling Technology, Cambridge, UK), pAkt (Ser 473) (Cell Signaling Technology, Cambridge, UK), pJNK (G-7) (sc-6254, Santa Cruz Biotechnology, CA), JNK (Cell Signaling Technology, Cambridge, UK) and GAPDH (FL-335) (Santa Cruz Biotechnology, CA) were utilized. As secondary antibodies we used goat anti-rabbit IgG-HRP (Santa Cruz Biotechnology, CA) and donkey anti-goat IgG-HRP (Santa Cruz Biotechnology, CA). The FDA approved drug library was obtained from Enzo Life Sciences (Farmingdale, NY).

### Cell Culture Studies

We used human umbilical vein endothelial cells (HUVECs) in passage 3–5. Cells were isolated from human umbilical veins with the help of Heat-inactivated Fetal Bovine Serum (FBS) (Thermo Fisher Scientific, Waltham, MA), Phosphate-buffered saline (Thermo Fisher Scientific, Waltham, MA), Trypsin/Ethylenediaminetetraacetic acid solution (Biochrom, Berlin, Germany) and Collagenase (Biochrom, Berlin, Germany) and were cultured in endothelial cell growth medium containing 2% FBS according to the manufacturer’s instructions (Lonza, Basel, Switzerland). Informed consent was obtained from all HUVEC donors and approved from the ethical committee of Hannover Medical School (Nr. 1303–2012). Cell isolation was conducted in accordance with institutional and governmental guidelines. Unless otherwise stated, HUVECs were stimulated with 10 ng/mL recombinant human TNF-alpha (R&D Systems, Minneapolis, MN). 1 mM Wortmannin (Sigma-Aldrich, St. Louis, MO), 10 ng/ml Interleukin-1β (Life Technologies, Carlsbad, CA), 50 μM LY294002 (Cell Signaling Technology, Cambridge, UK), 100 ng/mL Phorbol-12-myristate-13-acetate (PMA) (Merck Millipore, Darmstadt, Germany), 10 μM Flunarizine dihydrochloride (Sigma-Aldrich, St. Louis, MO), 10 μM Amlodipine besylate (Sigma-Aldrich, St. Louis, MO), 10 μM Mibefradil dihydrochloride hydrate (Sigma-Aldrich, St. Louis, MO), 50 μM TTA-A2 (Sigma-Aldrich, St. Louis, MO), 1 μM Thapsigargin (Santa Cruz Biotechnology, CA), 10 μM Ethylenediaminetetraacetic (Sigma-Aldrich, St. Louis, MO) and DMSO for molecular biology (Sigma-Aldrich, St. Louis, MO). Specific siRNA against Angpt-2 and Tie-2 (both customized from Silence Therapeutics, Berlin, Germany) were transfected with serum free medium (Lonza, Basel, Switzerland) and lipofectamine RNAiMAX Reagent (Thermo Fisher Scientific, Waltham, MA). As control siRNA we used an oligonucleotide without homology to any known mammalian gene. Results were reproduced n = 4–7 times per condition.

### Mouse Studies

All experiments were approved by the local authorities at Hannover Medical School and conducted in accordance with institutional and governmental guidelines (LAVES Lower Saxony, Ref Nr. 12/0681). Eight to 12 weeks old C57BL/6 J male mice were purchased from Charles River (Sulzfeld, Germany) and the Central Animal Facility of Hanover Medical School. For 3 days the mice received 25 mg/kg bodyweight (bw) of Flunarizine dihydrochloride (Sigma-Aldrich, St. Louis, MO) or a vehicle control orally once a day. On the third day, 17.5 mg/kg bw lipopolysaccharide (LPS) from E. coli serotype O111:B4 (Sigma-Aldrich, St. Louis, MO) was administered intraperitoneally (i.p.). After 12 hrs the mice were sacrificied for organ harvest and further molecular analysis. For survival studies, mice received 25 mg/kg bw Flunarizine or vehicle control orally for three days and were injected with 20 mg/kg bw LPS on the third day.

### Enzyme-linked immunosorbent assay (ELISA)

The Angpt-2 concentration in cell culture supernatant or cell lysates was measured with a commercial human Ang2 DuoSet ELISA (DY628, R&D Systems, Minneapolis, MN). Except for normal mouse serum (Jackson ImmunoResearch Laboratories, Westgrove, PA) and Bovine Serum Albumin (BSA) (Sigma-Aldrich, St. Louis, MO) the required solutions were purchased from R&D Systems. In order to detect the circulating Angpt-2 concentration from murine blood an ELISA Kit for Angpt-2 from USCN (Wuhan, China) was used.

### Fluorescent Immunocytochemistry

Coverslips were coated with collagen (Sigma-Aldrich, St. Louis, MO), and HUVECs were grown to confluency on them. At the end of the experiment, cells were fixed with 4% Paraformaldehyde, blocked with 10% donkey serum (Jackson Immuno Research Inc., West Grove, PA) and permeabilized with 0.1% Triton X-100 in PBS (Sigma-Aldrich, St. Louis, MO). Afterwards the primary antibody incubated for 1 h at room temperature. In order to visualize the binding of the primary antibody, cells were further incubated with fluorescent secondary antibodies for 1 h. As a secondary antibody Alexa Fluor 555 donkey anti goat IgG (Thermo Fisher Scientific, Waltham, MA) was used. 4′,6-diamidino-2-phenylindole (DAPI) was purchased from Sigma-Aldrich (St. Louis, MO). Finally the coverslips were affixed with Aqua-Poly/Mount (Polysciences Inc., Eppelheim, Germany). The images were taken with a Leica DMI 6000B microscope and were obtained with the same gain and offset conditions.

### Fluorescent Immunohistochemistry

Cryosections (6 μm) were blocked with 10% donkey serum (Dianova) and stained with primary antibodies against Gr-1 and an endothelial specific lectin (LEA-1).

### RNA Isolation and Quantitative (q) PCR

The RNeasy Mini Kit (Qiagen, Hilden, Germany) was used to extract total RNA from organ tissue and cultured cells per manufacturers’ instructions. Via the Transcriptor First Strand cDNA Synthesis (Roche Diagnostics, Rotkreuz, Switzerland) 1 μg of total RNA was then reverse transcribed to cDNA followed by SYBR Green real-time-quantitative PCR using a LightCycler 480 II (Roche, Basel, Switzerland). The following primers were used for the quantification: murine β-Actin (Fw: CCT GAG CGC AAG TAC TCT GTG T; Rev: CTG CTT GCT GAT CCA CAT CTG), murine Tie2 (Fw: ACT CTT CAT GTA CAA CGG CCA TT; Rev: AGT GGG TGG CTT GCTT GGT A), murine Angpt-2 (Fw: GCT GAA GGA CTG GGA AGG C; Rev: GGA CTC TTC ACC AGC GAG GTA), murine ICAM (Qiagen, Hilden, Germany), murine TNFα (Qiagen, Hilden, Germany), murine IL6 (Qiagen, Hilden, Germany), human β-Actin (Fw: CTG GAA CGG TGA AGG TGA CA; Rev: AGT CCT CGG CCA CAT TGT G), human Tie2 (Fw: CAG TAC GTG GTC CGA GCT AGA GT; Rev: TAA GGG TCC AAG CAG TGA GAT CT), human Angpt-2 (Fw: GCC GCT CGA ATA CGA TGA CT; Rev: GCT TCA TTA GCC ACT GAG TGT TGT). For each sample Triplicate RT-qPCR analyses were performed, and the mean of the obtained threshold cycle values (CT) was taken. Gene expression was standardized to the expression of the housekeeping gene, yielding the ∆CT value.

### Western Blot Analysis

After washing with PBS (Sigma-Aldrich, St. Louis, MO), radioimmunoprecipitation assay buffer (including 1 mM Na3VO4, 50 mM NaF, protease inhibitors [Roche Diagnostics, Mannheim, Germany]) was used to lyse the cells. The lysate was centrifuged at 4 °C for 15 minutes at 12000 rpm. Supernatant was collected and its concentration was measured with a Pierce BCA Protein Assay Kit (Thermo Scientific, Rockland, IL). Proteins were separated by SDS polyacrylamide gel electrophoresisa, transferred to PVDF (polyvinylidene fluoride) membranes (Merck Millipore, Darmstadt, Germany) and incubated with appropriate antibodies. The first antibody was incubated overnight at 4 °C, while the incubation of the second antibody was performed for 1 h at room temperature. Washing steps of the membrane were performed with TBST (20 mM Tris, 150 mM NaCl, 0.1% Tween20 [Merck]). To detect the proteins, secondary antibodies conjugated to horseradish peroxidase (Santa Cruz Biotechnology, CA) were utilized, the chemiluminescence was enhanced with SuperSignal™ West Pico Chemiluminescent Substrate (Life Technologies, Carlsbad, CA) and the signal was detected with Versa Doc Imaging System Modell 3000 (BioRad, Hercules, CA).

### Transendothelial Electrical Resistance (TER)

TER was measured using an electric cell-substrate impedance sensing system (Applied BioPhysics Inc.). Values were pooled at discrete time points and plotted versus time. Each condition’s end point resistance was divided by its starting resistance to give the normalized TER. Confluence was determined by the monolayer achieving manufacturer-recommended electric criteria.

### Live cell imaging

Cells were grown to confluency on an open μ-Slide with 4 wells (Ibidi, Munich, Germany). In order to visualize changes in intracellular calcium cells were incubated in 1 mM Fluo-4 AM (Life Technologies, Carlsbad, CA), a labeled calcium indicator, for 30 minutes. After washing with a bathing Solution (10 mM 100 g/L D-(+)-Glucose solution [Sigma Aldrich], 10 mM N-(2-Hydroxyethyl)piperazine-N′-(2-ethanesulfonic acid) solution [Sigma Aldrich], 5 mM KCl [AppliChem], 1 mM MgCl_2_ [Merck], 2 mM CaCl_2_ [Merck], Ampuwa solution [Fresenius Kabi]) cells had to sit in bathing solution for further 30 minutes before the actual stimulation. Images of two different positions for each well were taken every 60 seconds with an Axio Observer.Z1 Zeiss microscope.

### Statistical Analyses

Columns are presented as mean ± SEM. Two-tailed p values of less than 0.05 were considered to indicate statistical significance. Mann-Whitney U test was utilized in order to compare different groups. Whenever more than two groups have been compared we performed multiple corrections with one-way ANOVA (Kruskal-Wallis test) followed by non-parametric Dunn’s post-hoc analysis. Survival data were analyzed by log-rank test and visualized by Kaplan-Meier curves. We used GraphPad Prism 5 (La Jolla, CA) for data analysis and graph generation.

## Additional Information

**How to cite this article:** Retzlaff, J. *et al*. Flunarizine suppresses endothelial Angiopoietin-2 in a calcium - dependent fashion in sepsis. *Sci. Rep.* **7**, 44113; doi: 10.1038/srep44113 (2017).

**Publisher's note:** Springer Nature remains neutral with regard to jurisdictional claims in published maps and institutional affiliations.

## Supplementary Material

Supplementary Information
