# Supplementary material for: Flunarizine suppresses endothelial Angiopoietin-2 in a calcium - dependent fashion in sepsis
Source: Sci Rep. 2017 Mar 9;7:44113. doi: 10.1038/srep44113 (PMC5343493; doi:10.1038/srep44113)
Supplement: Supplementary Information [file srep44113-s1.pdf]

SUPPLEMENTAL MATERIAL

Flunarizine suppresses endothelial Angiopoietin-2 in a calcium - dependent fashion in sepsis

Jennifer Retzlaff[1,+], Kristina Thamm[1,+], Chandra C Ghosh[2], Wolfgang Ziegler[3], Hermann Haller[1], Samir M Parikh[2], Sascha David[1]

[1] Division of Nephrology and Hypertension, Medical School Hanover, Germany

[2] Beth Israel Deaconess Medical Center and Harvard Medical School, Center for Vascular Biology Research, Boston, MA, USA

[3] Department of Pediatric Kidney, Liver and Metabolic Diseases, Hannover Medical School

[+] these authors contributed equally to this work

Correspondence to:

Sascha David, MD

Medical School Hannover, Department of Nephrology & Hypertension

Carl-Neuberg-Str. 1, 30625 Hannover, GERMANY

Phone (+49) - 511 5326319 Fax (+49) 511 - 55 23 66

E-mail: david.sascha@mh-hannover.de

Supplemental Figures

Supplemental Figure 1

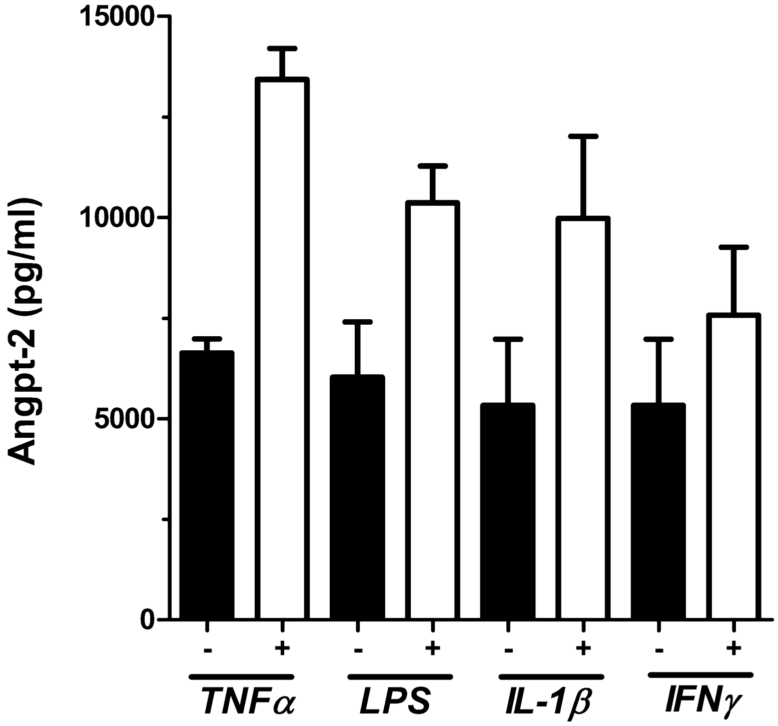

Evaluation of Angiopoietin-2 (Angpt-2) upon stimulation with different sepsis mediators.

Human umbilical vein endothelial cells (HUVECs) were either stimulated with 10ng/mL TNFα (+), 100ng/mL LPS (+), 10ng/mL IL-1β (+), 10ng/mL IFNγ (+) or control (-) for 24 hrs and Angpt-2 concentration in the supernatant was measured by ELISA (n=2).

Supplemental Figure 2

SF 2A Flunarizine decreases Angiopoietin-2 (Angpt-2) transcript expression upon stimulation.

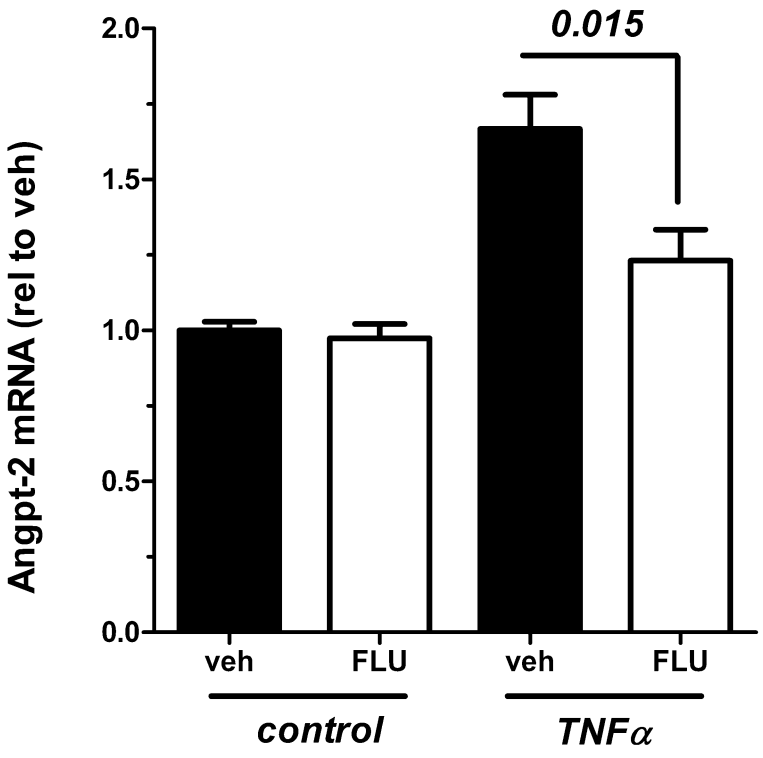

Real-time polymerase chain reaction (RT-PCR) for Angpt-2 in human umbilical vein endothelial cells (HUVECs) stimulated with 10 ng/mL TNFα or control for 2 hrs after pretreatment with either Flunarizine (FLU) or vehicle for 1 h (n=6). Columns are presented as mean ± SEM.

SF 2B Flunarizine reduced TNF downstream signaling.

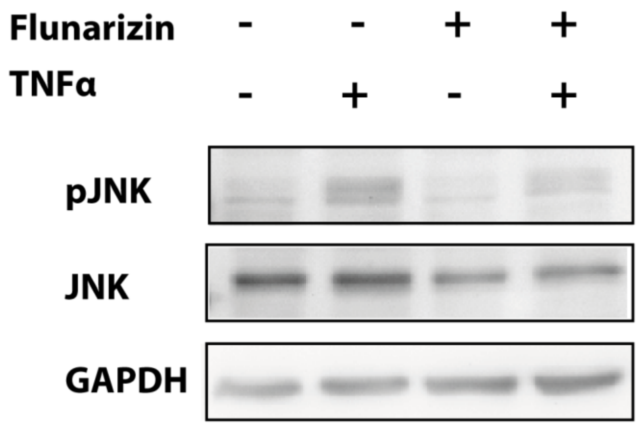

Immunoblot for phospho(p) and total JNK from human umbilical vein endothelial cell (HUVEC) lysates stimulated with 10 ng/mL TNFα or control for 0.5 hrs after pretreatment with either Flunarizine (FLU) or vehicle for 1h (n=2).

SF 2C IL-1ß induced Angpt-2 increase is reduced by Flunarizine.

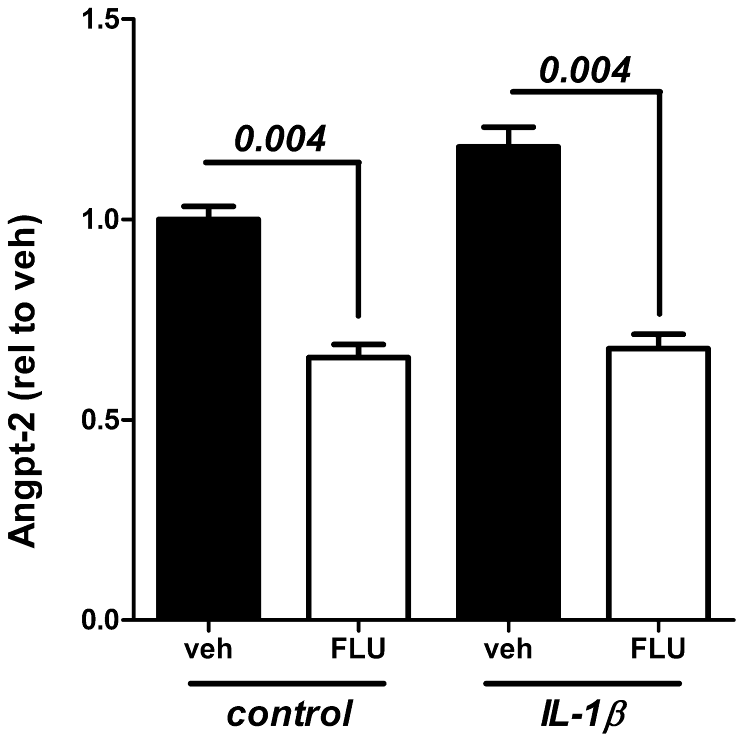

ELISA for Angpt-2 in the supernatant of HUVECs stimulated with 10 ng/mL Interleukin (IL)-1ß or control for 12 hrs after pretreatment with either Flunarizine (FLU) or vehicle for 1 h (n=6). Columns are presented as mean ± SEM.

Supplemental Figure 3

SF 3A Flunarizine does not require Tie2 to reduce Angpt-2.

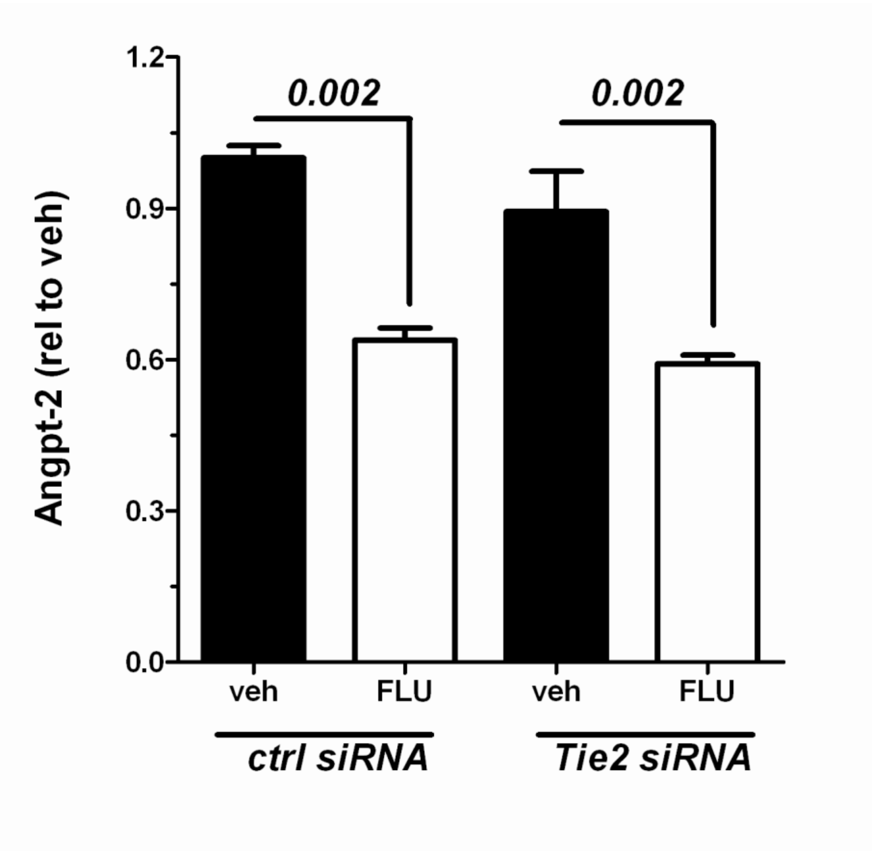

Human umbilical vein endothelial cells (HUVECs) were treated with 10µM Flunarizine (FLU) or vehicle for 6 hrs after transfection with control siRNA or Tie2 siRNA and the concentration of Angpt-2 in the supernatant was determined by ELISA (n=4-6). Columns are presented as mean ± SEM.

SF 3B Wortmannin inhibits phosphorylation of Akt.

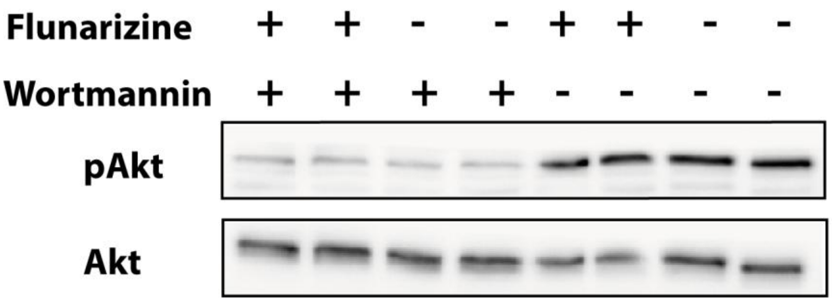

HUVEC lysates from cells pretreated with 1µM Wortmannin or control for 1h and stimulated with 10µM Flunarizine (FLU) or vehicle for 12 hrs were immunoblotted with Anti-pAkt, Anti-Akt and Anti-GAPDH as a loading control (n=4).

SF 3C Flunarizine reduces Angpt-2 independently from PI3K/Akt downstream of Tie-2.

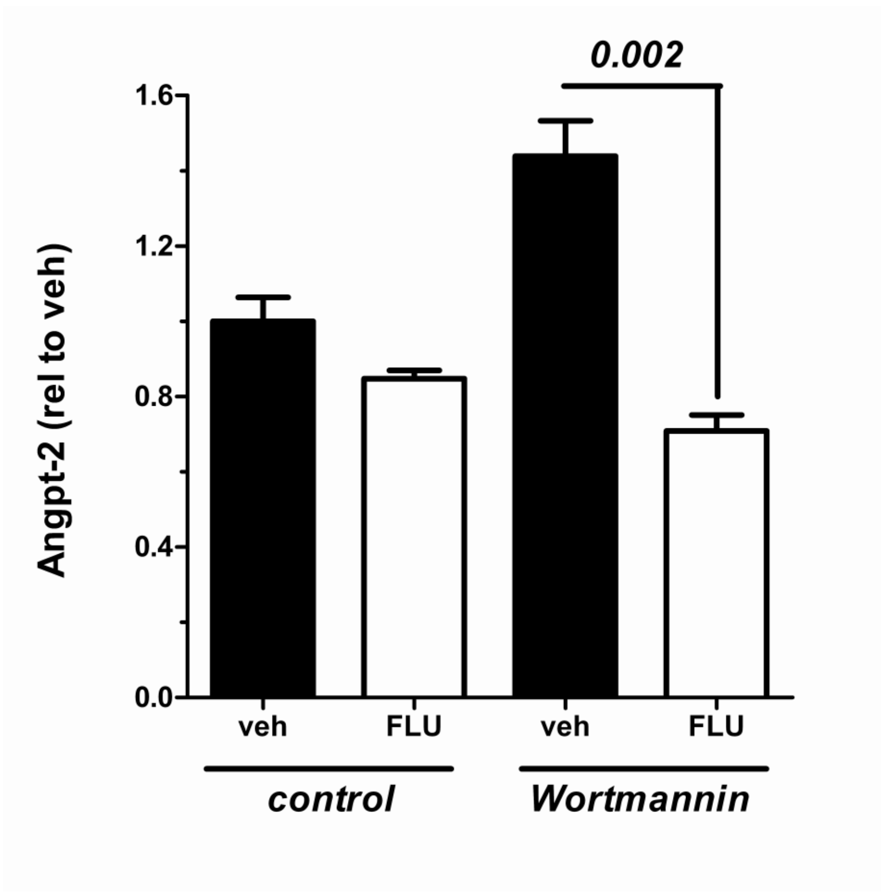

Angpt-2 concentration in the supernatant of cells pretreated with 1µM Wortmannin or control for 1h and stimulated with 10µM Flunarizine (FLU) or vehicle for 6 hrs was measured by ELISA (n=6). Columns are presented as mean ± SEM.

SF 3D Flunarizine does not activate Tie2 nor downstream AKT.

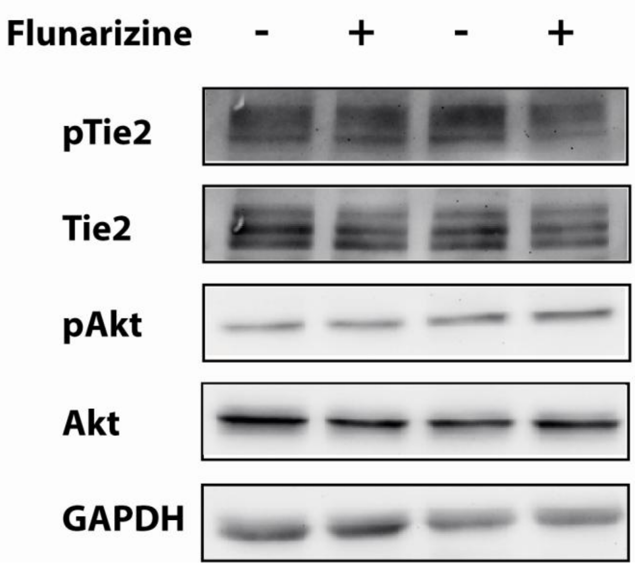

Immunoblot for Akt, pAKT, Tie2 and pTie2 and GAPDH as a loading control from HUVECs treated with 10µM Flunarizine or control for 18 hrs (n=3).

Supplemental Figure 4

SF 4A PMA increases Angpt-2.

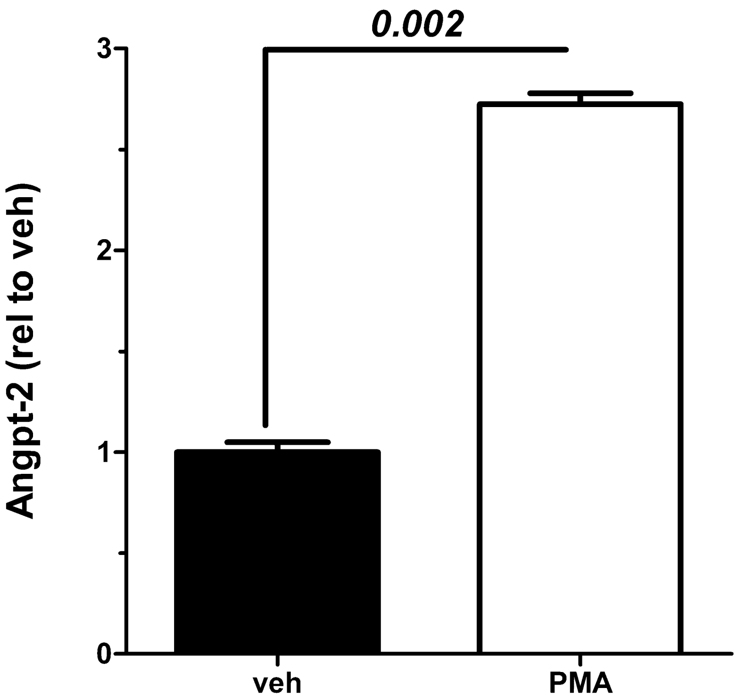

For 24 hrs HUVECs were stimulated with 100ng/mL Phorbol-12-myristat-13-acetat (PMA) or vehicle and Angpt-2 in the supernatant was quantified by ELISA (n=6). Columns are presented as mean ± SEM.

SF 4B Role of Calcium in Flunarizine-dependent Angiopoietin-2 (Angpt-2) regulation

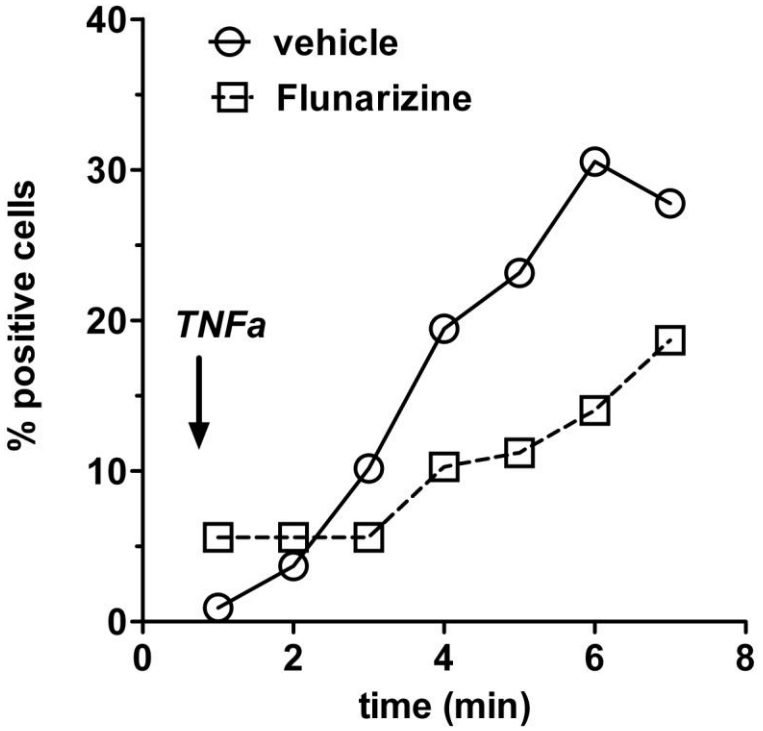

Live cell imaging with the calcium indicator Fluo-4 in HUVECs who were pretreated with 10µM Flunarizine or vehicle and stimulated with 10ng/mL TNFα (n=1). Results are shown as percentage of Fluo-4 positive cells per time.

Supplemental Figure 5

Flunarizine does not lower Angiopoietin-2 (Angpt-2) mRNA in the kidney.

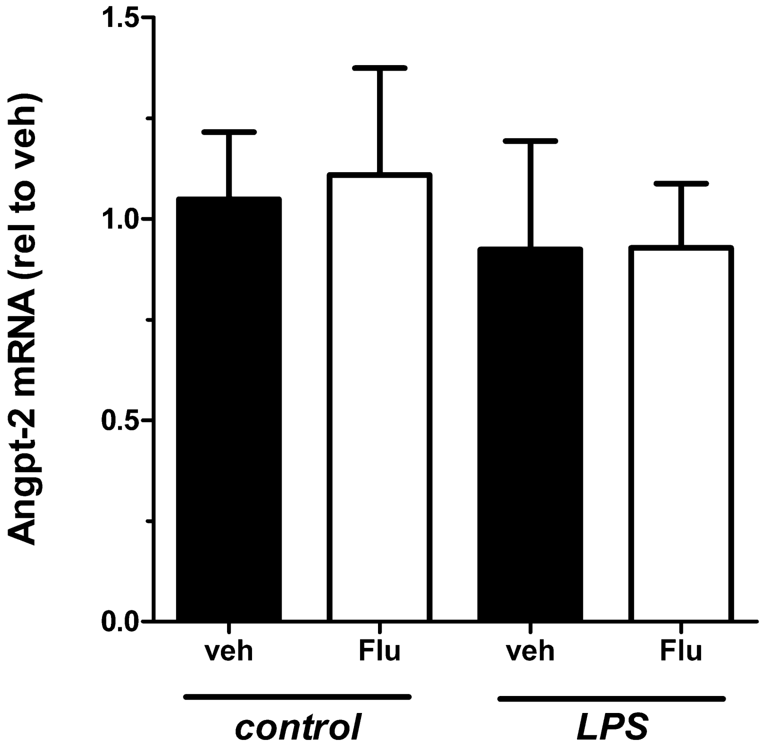

Real-time polymerase chain reaction (RT-PCR) from kidney homogenates for Angpt-2 (n=3-8).
